# What informs a one-party state’s foreign policy? The prospects of a neoclassical realism interpretation of Laos’ ASEAN Chairmanships

**DOI:** 10.12688/f1000research.176844.1

**Published:** 2026-01-21

**Authors:** Bama Andika Putra

**Affiliations:** 1 University of Bristol School of Sociology Politics and International Studies, Bristol, England, UK; 2 Department of International Relations, Universitas Hasanuddin, Indonesia, Indonesia

**Keywords:** ASEAN, Laos, Neutrality, Foreign Policy, Southeast Asia

## Abstract

How do state actors act in regional organizations? For decades, international relations scholars assessing Southeast Asia have examined the agency of middle powers in the region, exerting influence and shaping the introduction of regional norms. However, less has been assessed in the context of smaller states such as Laos, which has displayed some unique traits in its foreign policy. Among the empirical anomalies is Laos’ display of neutrality, despite growing closer to China’s lucrative economic opportunities in the past decades. As a means to understand why Laos’s chairmanship roles have displayed neutrality, this study bridges the relevance of neoclassical realism’s theoretical framework to make sense of foreign policies that are out of the ordinary. Drawing on primary and secondary data on Laos’ ASEAN chairmanships in 2016 and 2024, this study argues that domestic considerations (the growing negative sentiment towards economic ties with China) and external determinants (power relations with China and ASEAN regionalism) affect Laos’ external outlook.

## 1. Introduction: The Puzzle to Laos’ ASEAN Chairmanships

Laos has held the chairmanship role of the Association of Southeast Asian Nations (ASEAN) three times since its membership in the organization in 1997. ASEAN is a unique regional organization, prioritizing the importance of what scholars and observers name as the ‘ASEAN Way,’ with its diplomatic conduct geared to the non-coercive resolution of conflicts, consensus-based decision-making, and non-interference of the domestic politics of its member states ( Narine, 1997; Caballero-Anthony, 2005; Beeson, 2009; Tekunan, 2015; Darwis, Putra and Cangara, 2020). In Laos’ chairmanships in 2016 and 2024, Laos displayed its neutrality, aiming to take the middle pathway in tensions that have divided the ASEAN members, such as the Indo-Pacific great power rivalry ( Saha, 2018; Tertia and Perwita, 2018; Medcalf, 2019; Mubah, 2019; Wheeler, 2020), and the South China Sea dispute ( Fravel, 2011; Thayer, 2011; Blazevic, 2012; Yahuda, 2013; Putra, 2020, 2022, 2023b; Nguyễn Anh, 2023). As a small, one-party, landlocked Southeast Asian state, how can we make sense of Laos’ neutrality?

Concerning China’s Belt and Road Initiative (BRI), the expectation is that Laos would side with China in ASEAN. Studies have already concluded that Laos is a ‘vassal’ or ‘satellite’ nation to China ( Hunt, 2016; Kuik, 2021; Macan-Markar, 2022; Lin, 2023a; Mahtani and Huiying, 2024). China’s total investments in Laos from 2005 until 2024 accumulates to USD 16.5 Billion ( AEI, 2024). Several of the large-scale infrastructure development projects that Chinese investments have helped build since the launch of the BRI in 2013 include the Vientiane-Boten railway, special economic zones, and hydropower dams ( SIIS, 2017; Kuik, 2021; LMOFA, 2021; Seneviratne, 2024). Consequently, scholars have argued a strong connection between China’s BRI and the survival of the Lao People’s Revolutionary Party (LPRP) regime ( Lampton, Ho and Kuik, 2020; Kuik, 2021; Kuik and Rosli, 2023). In contrast to Laos’ condition, Cambodia’s ASEAN chairmanship in 2012 aligned with China’s national interests regarding ASEAN’s response towards the South China Sea ( Minh Vu, 2019; Dunst, 2021; Pich, 2021). So why doesn’t Laos display a similar gesture?

This opinion article argues that consultation is needed for the international relations theory of neoclassical realism to make sense of such an empirical anomaly. Neoclassical realism aims to make sense of foreign policy choices that are out of the ordinary ( Rose, 1998; Schweller, 2004; Kitchen, 2010; Foulon, 2015; Kozub-Karkut, 2019). Utilizing primary and secondary data related to Laos’ ASEAN chairmanship in 2016 and 2024, this descriptive study argues the relevance of domestic considerations (such as growing negative sentiments towards economic ties with China) and external influences (power relations with China and ASEAN regional integration) that affect its foreign policy.

## 2. Interpretations of Laos’ international relations and the potential of the neoclassical realism framework

Currently, no study has examined Laos’ engagement with ASEAN. The dominant discourse assessing Laos’ external outlook has focused on interpreting Laos’ growing ties with China in the economic domain. The discourse itself is divided into two groups, with those arguing that Laos adopts a balancing strategy vis-à-vis China ( Hunt, 2016; Sims, 2021; Lin, 2023a, 2023b) and those that argue that Laos is approaching the status of becoming a ‘vassal’ state to China ( Tuo, Hui and Zhongxia, 2018; Macan-Markar, 2022; Mahtani and Huiying, 2024; Sayalath, 2024; Walker, 2024). Nevertheless, Laos’ foreign policy is not only confined to its relations with its neighbor to the North, as studies have discussed Laos’ active engagements with its regional neighbors such as Thailand and Vietnam ( Thayer, 1982; Giang and Phuong, 2024; Phoonphongphiphat, 2024), as well as with the US ( Sayalath, 2024). Meanwhile, with Laos’ policies in ASEAN, there have only been media reports on Laos’ neutrality throughout its chairmanship ( Patton, 2024; Sims, 2024). Therefore, in its current form, Laos’ neutrality is ‘taken for granted.’

Neoclassical realism bridged to this study argues the relevance of two variables. First, the ‘systemic stimuli’ is its independent variable. The argument is that states are primarily influenced by their external environment, limiting the number of foreign policy options a state can take ( Foulon, 2015; Ripsman, Taliaferro and Lobell, 2016). It is a state-centric conception ( Rose, 1998), with influencing sub-variables related to “power and position in the international system and by its relative share of material capabilities” ( Ripsman et al., 2016, p.56). This opinion article will consider the sub-variables founded by Norrin Ripsman, Jeffrey Taliaferro, and Steven Lobell’s ‘Neoclassical Realist Theory of International Politics’: power and position in the international system, the relative share of material capabilities, structural modifiers, clarity, and strategic environment ( Ripsman, Taliaferro and Lobell, 2016).

The intervening variable is understood as the unit and sub-unit level variables that are influential in informing a state’s foreign policy. These include perception, decision-making, and policy implementation processes, which are constructed by leader images, strategic culture, state-society relations, and domestic institutions ( Ripsman, Taliaferro and Lobell, 2016). As intervening variables, these act as filters in perceptions and actions that complement a country’s response to a systemic stimulus.

The case of Laos is particularly interesting for this assessment, as Laos’ governance is confined and limited to the authority of the LPRP as the dominant and only political party of Laos. As one of the few states that openly endorse communism and adopt authoritarian rule, its actions in ASEAN could, with ease, represent the national interests of China to secure more funding for its ambitious development plans ( Stuart-Fox, 1998; Cuyvers, 2019; Atkinson, 2021). Nevertheless, its consideration of the possible consequences of this shows the potential relevance of intervening variables that fuel Laos’ foreign policies.

## 3 Deciphering Laos’ neutrality in ASEAN: The role of systemic stimuli and internal filters in Laos’ external policies

Laos’ ASEAN chairmanship in 2016 and 2024 faced several challenges that had its roots with China’s involvement in the region. By 2016, China’s maritime diplomatic strategies had evolved to a level that was perceived as assertive by claimant states to the South China Sea, the Philippines, Vietnam, Brunei Darussalam, and Malaysia ( Reuters, 2016; Yu, 2016; Basawantara, 2020; Chubb, 2022; Putra and Cangara, 2022; Putra, 2023a). The deepened great power politics in the Indo-Pacific also brought into question the possible fading role of ASEAN in the Indo-Pacific region as great powers started to establish their groupings to define, in their terms, the geopolitical significance of the region ( Chacko and Willis, 2018; Scott, 2019; Singh and Tsjeng, 2020; Tan, 2020; Hall, Lee-Brown and Strating, 2024).

In 2016, Laos displayed neutrality amid tensions between the Philippines and China over the language used for the 2016 Joint Statement of ASEAN Foreign Ministers and China. The Philippines, being a victim of China’s assertive claiming within its Exclusive Economic Zone, demanded the chair to include legal phrases in the joint statement; meanwhile, China preferred the adoption of vague languages ( Odgaard, 2003; Sayalath and Creak, 2017; Storey, 2018; Hu, 2021; Kittikhoun, 2022; Sayalath, 2024). Laos chose the middle pathway and decided to make both parties equally unhappy by refraining from facilitating both interests ( Lin, 2023b). As one of the joint statement’s operative clauses mentioned: “The parties reaffirm their respect for and commitment to the freedom of navigation in and overflight above the South China Sea as provided for by the universally recognized principles of international law” ( ASEAN, 2016, p.2).

Meanwhile, Laos’ chairmanship in 2024 emphasized the importance of ASEAN centrality vis-à-vis the tensions in the Indo-Pacific. As stated in the 44 ^th^ and 45 ^th^ ASEAN Summit Chairman’s Statement, Laos reiterated the relevance of the ASEAN Outlook on the Indo-Pacific (AOIP) for the region by reaffirming “ASEAN’s commitment to promote an enabling environment for peace, stability, and prosperity in the region by leading the evolving regional architecture including through ASEAN-led mechanisms and managing the impact of geopolitical and geostrategic shifts …” ( ASEAN, 2024, p.10). With regards to the South China Sea dispute, Laos again refrained from siding with China by emphasizing the importance of an ASEAN-centered solution and parties to avoid confrontational actions: “We emphasized the importance of self-restraint in the conduct of all activities by claimants and all other states […] and thus welcomed practical measures that could reduce tensions and the risks of accidents, misunderstandings, and miscalculations” ( ASEAN, 2024, p.41).

The international system does not generate a fixed signal. China’s rise in the Asia Pacific exerts pressure on smaller states like Laos to align with China to secure economic benefits. However, this is not an automatic process. Based on the strategic environment of Laos, there is still a special perception reserved for Thailand and Vietnam ( Thayer, 1982; Giang and Phuong, 2024; Phoonphongphiphat, 2024) due to historical attachments, convergence of political views and mutual economic benefits. Therefore, fully siding with China will expose Laos to self-isolation and an overly dependent foreign policy. For Laos, over-dependence on a great power is dangerous for the stability of the state, as seen with Laos’ struggle during the Cold War after the weakening and eventual collapse of the Soviet Union ( Meng, 1987; Evans, 1998; Rathie, 2017; Leon, 2024).

Structural modifiers are another systemic stimuli variable relevant to Laos’ actions in its ASEAN chairmanships. Laos is a landlocked country, which ultimately means that its options for development are severely limited. Laos perceives that it is pivotal to diversify its economic relations with the Southeast Asian states by displaying good faith in ASEAN, as it helps accelerate Laos’ integration into the Southeast Asian markets. In its current form, Laos is categorized into the CMLV (Cambodia, Myanmar, Laos, Vietnam) category, a list of the least-developed economies in Southeast Asia ( Stuart-Fox, 1998; Chirathivat, 2002; Narjoko and Amri, 2007; Cuyvers, 2019). A display of commitment towards the ASEAN Way allows Laos to open up alternative means of cooperation. The economic ties between Laos and China have negatively impacted Laos’ foreign debt rates, which are currently higher than a hundred percent of its GDP ( Macan-Markar, 2022; Tiwari, 2024; Walker, 2024).

What can the independent variables of neoclassical realism explain concerning Laos’ neutrality in its 2016 and 2024 ASEAN chairmanship? China’s investments in Laos have started to generate negative responses from the Lao people. The practices of shoddy constructions, forced land grabbing, granting of land and mining concessions, an increase in debt, and a larger presence of Chinese working migrants have all contributed to the rise of this negative perspective ( Hunt, 2016; Tuo, Hui and Zhongxia, 2018; Sims, 2020, 2021; Kuik, 2021). Comparing the responses between the 2019 and 2024 surveys, Yusof-Ishak Institute’s ‘State of Southeast Asia’ reported that there is an increase in negative perceptions of the Lao people towards China’s investments within the country, and the increasing harm that directly affects the citizens ( ISEAS, 2019, 2024). Assessing the unit and sub-unit variables of neoclassical realism poses one main challenge. Unlike democratic settings, there is practically no room for other voices of opposition to establish change within Laos’ governance due to its autocratic rule. Therefore, the LPRP holds the ultimate rule in the perception, decision-making, and policy implementation of its foreign policies.

Nevertheless, accommodating the growing voices to distance Laos from a dependent economic tie with China is heard for the LPRP. As studies in the past have shown, even autocratic states consider the voices of their opposition. As seen in the case of Cambodia, Hun Sen’s nearly three-decade rule had to start accommodating the concerns of opposition movements with the rise of Cambodia’s opposition political party’s popularity ( Blanchard, 2017; Morgenbesser, 2019; Thul, 2023). Bader found that autocratic nations must display strong economic performances to complement the deficiencies in their political participation ( Bader, 2015). However, if a growing opposition movement or voices are observed, the risks could be detrimental to the survival of a regime.

Therefore, in the case of the LPRP, the state-society relations sub-unit variable under neoclassical realism is influential in understanding Laos’ accommodation of the negative sentiment opinions within Laos. If the LPRP were to take the option of siding with China, as Cambodia did during its ASEAN chairmanship of 2012, it would risk the perception of growing dependent ties with China. The Lao people are increasingly cautious of China’s investments within the state, so this would not be a strategic policy for the LPRP. Practically all of China’s BRI projects in Laos have received backlash from the public. With the Vientiane-Boten railway project, for example, citizens questioned whether Laos was truly becoming the ‘hub’ of mainland Southeast Asian trade due to insufficient commodities to export ( Freeman, 2019; Seneviratne, 2024) and the fact that mining concessions were used as collateral in case the railway did not generate enough return for China’s investments ( Pang, 2017; SIIS, 2017; Albert, 2019; Walker, 2024). By taking the side of the ASEAN Way, the LPRP benefits from distancing itself from the discourse of being overly attached to China to satisfy the Lao people’s concerns over injustices associated with the BRI projects in Laos.

## Data Availability

No data are associated with this article.
