# What informs a one-party state’s foreign policy? The prospects of a neoclassical realism interpretation of Laos’ ASEAN Chairmanships

**DOI:** 10.12688/f1000research.176844.2

**Published:** 2026-03-13

**Authors:** Bama Andika Putra

**Affiliations:** 1 University of Bristol School of Sociology Politics and International Studies, Bristol, England, UK; 2 Department of International Relations, Universitas Hasanuddin, Indonesia, Indonesia

**Keywords:** ASEAN, Laos, Neutrality, Foreign Policy, Southeast Asia

## Abstract

How do state actors act in regional organizations? For decades, international relations scholars assessing Southeast Asia have examined the agency of middle powers in the region, exerting influence and shaping the introduction of regional norms. However, less has been assessed in the context of smaller states such as Laos, which has displayed some unique traits in its foreign policy. Among the empirical anomalies is Laos’ display of neutrality, despite growing closer to China’s lucrative economic opportunities in the past decades. As a means to understand why Laos’s chairmanship roles have displayed neutrality, this study bridges the relevance of neoclassical realism’s theoretical framework to make sense of foreign policies that are out of the ordinary. Drawing on primary and secondary data on Laos’ ASEAN chairmanships in 2016 and 2024, this study argues that domestic considerations (the growing negative sentiment towards economic ties with China) and external determinants (power relations with China and ASEAN regionalism) affect Laos’ external outlook.

## 1. Introduction: The Puzzle of Laos’ ASEAN Chairmanships

Laos has held the Association of Southeast Asian Nations (ASEAN) chairmanship three times since joining the organization in 1997. ASEAN is a unique regional organization, prioritizing the importance of what scholars and observers name as the ‘ASEAN Way,’ with its diplomatic conduct geared to the non-coercive resolution of conflicts, consensus-based decision-making, and non-interference of the domestic politics of its member states ( Narine, 1997; Caballero-Anthony, 2005; Beeson, 2009; Tekunan, 2015; Darwis, Putra, and Cangara, 2020). In Laos’ chairmanships in 2016 and 2024, Laos displayed its neutrality, aiming to take the middle pathway in tensions that have divided the ASEAN members, such as the Indo-Pacific great power rivalry ( Saha, 2018; Tertia and Perwita, 2018; Medcalf, 2019; Mubah, 2019; Wheeler, 2020), and the South China Sea dispute ( Fravel, 2011; Thayer, 2011; Blazevic, 2012; Yahuda, 2013; Putra, 2020, 2022, 2023b; Nguyễn Anh, 2023). As a small, one-party, landlocked Southeast Asian state, how can we make sense of Laos’ neutrality?

Regarding China’s Belt and Road Initiative (BRI), the expectation is that Laos would align with China within ASEAN. Studies have already concluded that Laos is a ‘vassal’ or ‘satellite’ nation to China ( Hunt, 2016; Kuik, 2021; Macan-Markar, 2022; Lin, 2023a; Mahtani and Huiying, 2024). China’s total investment in Laos from 2005 to 2024 amounts to USD 16.5 billion ( AEI, 2024). Several large-scale infrastructure development projects that Chinese investments have helped build since the launch of the BRI in 2013 include the Vientiane-Boten railway, special economic zones, and hydropower dams ( Kuik, 2021; LMOFA, 2021; Seneviratne, 2024). Consequently, scholars have argued for a strong connection between China’s BRI and the survival of the Lao People’s Revolutionary Party (LPRP) regime ( Lampton, Ho and Kuik, 2020; Kuik, 2021; Kuik and Rosli, 2023). In contrast to Laos’ position, Cambodia’s ASEAN chairmanship in 2012 aligned with China’s national interests regarding ASEAN’s response to the South China Sea ( Minh Vu, 2019; Dunst, 2021; Pich, 2021). So why doesn’t Laos display a similar gesture?

This opinion article argues that consultation is needed for the international relations theory of neoclassical realism to explain such an empirical anomaly. Neoclassical realism aims to explain foreign policy choices that are out of the ordinary ( Rose, 1998; Schweller, 2004; Kitchen, 2010; Foulon, 2015; Kozub-Karkut, 2019). Utilizing primary and secondary data on Laos’ ASEAN chairmanship in 2016 and 2024, this descriptive study argues that domestic considerations (such as growing negative sentiment towards economic ties with China) and external influences (power relations with China and ASEAN regional integration) affect its foreign policy.

## 2. Interpretations of Laos’ international relations and the potential of the neoclassical realism framework

Currently, no study has examined Laos’ engagement with ASEAN. The dominant discourse assessing Laos’ external outlook has focused on interpreting its growing economic ties with China. The discourse itself is divided into two groups, with those arguing that Laos adopts a balancing strategy vis-à-vis China ( Hunt, 2016; Sims, 2021; Lin, 2023a, 2023b) and those that argue that Laos is approaching the status of becoming a ‘vassal’ state to China ( Tuo, Hui and Zhongxia, 2018; Macan-Markar, 2022; Mahtani and Huiying, 2024; Sayalath, 2024; Walker, 2024). Nevertheless, Laos’ foreign policy is not only confined to its relations with its neighbor to the North, as studies have discussed Laos’ active engagements with its regional neighbors, such as Thailand and Vietnam ( Thayer, 1982; Giang and Phuong, 2024; Phoonphongphiphat, 2024), as well as with the US ( Sayalath, 2024). Meanwhile, in ASEAN, there have been only media reports on Laos’ neutrality throughout its chairmanship ( Patton, 2024; Sims, 2024). Therefore, in its current form, Laos’ neutrality is ‘taken for granted.’

Neoclassical realism, as applied to this study, argues for the relevance of two variables. First, the ‘systemic stimuli’ are the independent variable. The argument is that states are primarily influenced by their external environment, limiting the number of foreign policy options a state can pursue ( Ripsman, Taliaferro, and Lobell, 2016). It is a state-centric conception, with influencing sub-variables related to “power and position in the international system and by its relative share of material capabilities” ( Ripsman et al., 2016, p. 56). This opinion article will consider the sub-variables founded by Norrin Ripsman, Jeffrey Taliaferro, and Steven Lobell’s ‘Neoclassical Realist Theory of International Politics’: power and position in the international system, the relative share of material capabilities, structural modifiers, clarity, and strategic environment ( Ripsman, Taliaferro, and Lobell, 2016).

The intervening variable is understood as the unit- and sub-unit-level variables that influence a state’s foreign policy. These include perception, decision-making, and policy implementation processes, which are shaped by leader images, strategic culture, state-society relations, and domestic institutions ( Ripsman, Taliaferro, and Lobell, 2016). As intervening variables, these act as filters in perceptions and actions that complement a country’s response to a systemic stimulus.

The case of Laos is particularly interesting for this assessment, as its governance is confined to the authority of the LPRP, the dominant and only political party in Laos. As one of the few states that openly endorse communism and adopt authoritarian rule, it could, with ease, represent China’s national interests in ASEAN to secure more funding for its ambitious development plans ( Stuart-Fox, 1998; Cuyvers, 2019; Atkinson, 2021). Nevertheless, its consideration of the possible consequences underscores the potential relevance of intervening variables that fuel Laos’ foreign policies.

## 3. Deciphering Laos’ neutrality in ASEAN: The role of systemic stimuli and internal filters in Laos’ external policies

Laos’ ASEAN chairmanship in 2016 and 2024 faced several challenges rooted in China’s involvement in the region. By 2016, China’s maritime diplomatic strategies had evolved to a level that was perceived as assertive by claimant states to the South China Sea, the Philippines, Vietnam, Brunei Darussalam, and Malaysia ( Reuters, 2016; Yu, 2016; Basawantara, 2020; Chubb, 2022; Putra and Cangara, 2022; Putra, 2023a). The deepened great power politics in the Indo-Pacific also brought into question the possible fading role of ASEAN in the Indo-Pacific region as great powers started to establish their groupings to define, in their terms, the geopolitical significance of the region ( Chacko and Willis, 2018; Scott, 2019; Singh and Tsjeng, 2020; Tan, 2020; Hall, Lee-Brown and Strating, 2024). However, as noted in several other past studies, there is a discourse showing that recently introduced initiatives such as the ASEAN Outlook on the Indo-Pacific (AOIP) have served as ASEAN s proactive geopolitical tool to shape Southeast Asia’s responses towards uncertainties deriving from great power tensions in alignment to the ASEAN Way ( Acharya, 2019; Anwar, 2020; Singh and Tsjeng, 2020; Putra, Cangara and Darwis, 2024). The AOIP, therefore, has served as a shield for ASEAN, protecting the regional organization from the intensification of the US-China rivalry through measures consistent with ASEAN’s principles.

In 2016, Laos remained neutral amid tensions between the Philippines and China over the language used in the 2016 Joint Statement of ASEAN Foreign Ministers and China. The Philippines, being a victim of China’s assertive claiming within its Exclusive Economic Zone, demanded that the chair to include legal phrases in the joint statement; meanwhile, China preferred the adoption of vague language ( Odgaard, 2003; Sayalath and Creak, 2017; Storey, 2018; Hu, 2021; Kittikhoun, 2022; Sayalath, 2024). Laos chose the middle path and decided to make both parties equally unhappy by refraining from favoring either interest ( Lin, 2023b). As one of the joint statement’s operative clauses mentioned: “The parties reaffirm their respect for and commitment to the freedom of navigation in and overflight above the South China Sea as provided for by the universally recognized principles of international law” ( ASEAN, 2016, p. 2).

Meanwhile, Laos’ chairmanship in 2024 emphasized ASEAN centrality amid tensions in the Indo-Pacific. As stated in the 44 ^th^ and 45 ^th^ ASEAN Summit Chairman’s Statement, Laos reiterated the relevance of the AOIP for the region by reaffirming “ASEAN’s commitment to promote an enabling environment for peace, stability, and prosperity in the region by leading the evolving regional architecture including through ASEAN-led mechanisms and managing the impact of geopolitical and geostrategic shifts …” ( ASEAN, 2024, p. 10). With regards to the South China Sea dispute, Laos again refrained from siding with China by emphasizing the importance of an ASEAN-centered solution and parties to avoid confrontational actions: “We emphasized the importance of self-restraint in the conduct of all activities by claimants and all other states […] and thus welcomed practical measures that could reduce tensions and the risks of accidents, misunderstandings, and miscalculations” ( ASEAN, 2024, p. 41).

The international system does not generate a fixed signal. China’s rise in the Asia-Pacific exerts pressure on smaller states like Laos to align with it to secure economic benefits. However, this is not an automatic process. Given the strategic environment of Laos, there remains a special perception reserved for Thailand and Vietnam ( Thayer, 1982; Giang and Phuong, 2024; Phoonphongphiphat, 2024) due to historical attachments, convergence of political views, and mutual economic benefits. With Vietnam, for example, the special relationship can be attributed to the shared communist ideology and close ties that were earlier established during the Cold War. Fully siding with China will expose Laos to self-isolation and an overly dependent foreign policy. For Laos, over-dependence on a great power is dangerous to the state’s stability, as seen in Laos’ struggle during the Cold War after the weakening and eventual collapse of the Soviet Union ( Meng, 1987; Evans, 1998; Rathie, 2017; Leon, 2024).

Structural modifiers are another systemic-stimuli variable relevant to Laos’ actions during its ASEAN chairmanships. Laos is a landlocked country, which ultimately means that its options for development are severely limited. Laos perceives it as pivotal to diversify its economic relations with Southeast Asian states by demonstrating good faith in ASEAN, as this helps accelerate Laos’ integration into Southeast Asian markets. In its current form, Laos is categorized as part of the CMLV (Cambodia, Myanmar, Laos, Vietnam) group, which comprises the least-developed economies in Southeast Asia ( Chirathivat, 2002; Narjoko and Amri, 2007; Cuyvers, 2019). A display of commitment to the ASEAN Way allows Laos to explore alternative avenues of cooperation. The economic ties between Laos and China have negatively affected Laos’ foreign debt, which is currently higher than 100% of its GDP ( Macan-Markar, 2022; Tiwari, 2024; Walker, 2024). Although the exact figures have been debated, there is still a strong acknowledgement that Laos’ debts is concerning.

What can the independent variables of neoclassical realism explain concerning Laos’ neutrality in its 2016 and 2024 ASEAN chairmanship? China’s investments in Laos have started to generate negative responses from the Lao people. The practices of shoddy constructions, forced land grabbing, granting of land and mining concessions, an increase in debt, and a larger presence of Chinese working migrants have all contributed to the rise of this negative perspective ( Hunt, 2016; Tuo, Hui and Zhongxia, 2018; Sims, 2020, 2021; Kuik, 2021). Comparing the responses between the 2019 and 2024 surveys, the Yusof-Ishak Institute’s ‘State of Southeast Asia’ reported an increase in negative perceptions of the Lao people towards China’s investments in the country and the increasing harm that directly affects citizens ( ISEAS, 2019, 2024). Assessing the unit and sub-unit variables of neoclassical realism poses one main challenge. Unlike in democratic settings, there is virtually no room for other voices of opposition to effect change within Laos’ governance due to its autocratic rule. Therefore, the LPRP holds the ultimate rule in the perception, decision-making, and policy implementation of its foreign policies.

Nevertheless, there is a chance for the LPRP to accommodate the growing voices calling for a distance from China’s economic dependence. For example, as a comparison, studies have shown that even autocratic states consider the voices of their opposition. As seen in Cambodia, Hun Sen’s nearly three-decade rule had to start accommodating the concerns of opposition movements as the popularity of Cambodia’s opposition political party rose ( Blanchard, 2017; Morgenbesser, 2019; Thul, 2023). Bader found that autocratic nations must display strong economic performance to compensate for deficiencies in political participation ( Bader, 2015). However, if a growing opposition movement or voices are observed, the risks could be detrimental to a regime’s survival.

Therefore, in the case of the LPRP, the state-society relations sub-unit variable under neoclassical realism is influential in understanding Laos’ accommodation of the negative sentiment opinions within Laos. If the LPRP were to choose to side with China, as Cambodia did during its ASEAN chairmanship in 2012, it would risk the perception of growing dependence on China. The Lao people are increasingly cautious of China’s investments within the state, so this would not be a strategic policy for the LPRP. Practically all of China’s BRI projects in Laos have faced public backlash. With the Vientiane-Boten railway project, for example, citizens questioned whether Laos was truly becoming the ‘hub’ of mainland Southeast Asian trade due to insufficient commodities to export ( Freeman, 2019; Seneviratne, 2024) and the fact that mining concessions were used as collateral in case the railway did not generate enough return for China’s investments ( Pang, 2017; SIIS, 2017; Albert, 2019; Walker, 2024). By taking the side of the ASEAN Way, the LPRP benefits by distancing itself from the discourse of being overly attached to China, thereby satisfying the Lao people’s concerns about injustices associated with the BRI projects in Laos.

## Data Availability

No data are associated with this article.
