# Supplementary material for: What informs a one-party state’s foreign policy? The prospects of a neoclassical realism interpretation of Laos’ ASEAN Chairmanships
Source: F1000Res. 2026 Apr 24;15:97. Originally published 2026 Jan 21. [Version 3] doi: 10.12688/f1000research.176844.3 (PMC13129525; doi:10.12688/f1000research.176844.3)
Supplement: Peer review history

# Reviewer report: Reviewer response for version 3

**DOI:** 10.5256/f1000research.198822.r479975

**Published:** 2026-05-15

**Authors:** William J. Jones

**Affiliations:** 1 Mahidol University International College, Nakhon, Thailand

For an opinion piece this is a very robust examination. The primary area which I feel could be strengthened in the analysis is by surveying the position of other ASEAN member states with regard to the SCS issue and AOIP. The neoclassical approach is fine but it is also open to a broader analysis which is inclusive of not only, China as a factor in Lao PDR decision-making.

 Lao PDR is a small country and it tends to adhere to ASEAN norms and sensibilities, always preferring a quiet and non-confrontational approach. It also does not take an active role in ASEAN affairs, trying to steer the organization but instead forging consensus. Laos is very much influenced by Thailand and Vietnam. Perhaps, not as much as China but this cannot be discounted. Comparatively, Laos acts much differently than say Cambodia even though economic and infrastructure influence is similar. If the author can address this by comparative approach or by integrating the perspectives and positions of these other ASEAN states that hold much influence in Laos I feel the opinion piece would be highly valid and provide richer insight.

Is the topic of the opinion article discussed accurately in the context of the current literature?

Yes

Are arguments sufficiently supported by evidence from the published literature?

Yes

Are all factual statements correct and adequately supported by citations?

Yes

Are the conclusions drawn balanced and justified on the basis of the presented arguments?

Yes

Reviewer Expertise:

NA

I confirm that I have read this submission and believe that I have an appropriate level of expertise to confirm that it is of an acceptable scientific standard.

# Reviewer report: Reviewer response for version 3

**DOI:** 10.5256/f1000research.198822.r478854

**Published:** 2026-04-29

**Authors:** Mansur Juned

**Affiliations:** 1 Universitas Pembangunan Nasional Veteran Jakarta, Jakarta, Special Capital Region of Jakarta, Indonesia

I appreciate the author efforts on revising the paper. Overall, it has met standard of a comprehensive, data based, opinion article on F1000. However there is one thing that should be revised to avoid information inaccuracy.

  Please delete or update the  sentence of "In its current form, Laos is categorized as part of

 the CMLV (Cambodia, Myanmar, Laos, Vietnam) group, which comprises the least-developed

 economies in Southeast Asia ( Chirathivat, 2002; Narjoko and Amri, 2007; Cuyvers, 2019)". While it could be accurate while those references were written, I believe the least developed countries in Southeast Asia in 2024-2026 are no longer involving Vientam. Please refer to the latest data from UNCTAD

Is the topic of the opinion article discussed accurately in the context of the current literature?

Yes

Are arguments sufficiently supported by evidence from the published literature?

Partly

Are all factual statements correct and adequately supported by citations?

Partly

Are the conclusions drawn balanced and justified on the basis of the presented arguments?

Yes

Reviewer Expertise:

Area of the research: Security Studies, Cybersecurity, Geopolitic in Indo-Pacific

I confirm that I have read this submission and believe that I have an appropriate level of expertise to confirm that it is of an acceptable scientific standard.

# Reviewer report: Reviewer response for version 2

**DOI:** 10.5256/f1000research.197347.r467547

**Published:** 2026-04-20

**Authors:** Quoc Dung Mai

**Affiliations:** 1 Ho Chi Minh City University of Industry and Trade, Ho Chi Minh City, Ho Chi Minh City, Vietnam

I acknowledge and appreciate the author’s efforts in incorporating and revising the manuscript based on feedback from the previous round. These revisions have helped make the structure of the paper clearer. However, upon careful review, I find that several important gaps in academic rigor and empirical data remain and must be thoroughly addressed before the manuscript can be accepted for Indexing. In particular, the author should further strengthen the following points:

 1. On macroeconomic data:

 Although some updates have been made, the assertion of an absolute figure “above 100%” in 2024 is not methodologically convincing. In scientific research, the use of a fixed figure while international organizations (such as IMF, World Bank, and AMRO) report differing estimates may lead to accusations of “data exaggeration” to support an argument. It is essential to cite data from at least two reputable financial institutions (e.g., Fitch, Statista, or IMF) to ensure objectivity and cross-verification. Acknowledging statistical discrepancies will make the argument more professional and more resilient to critique.

 2. On Laos’s role in the regional structure:

 The current argument that Laos is “actively shaping the regional order” remains vague and may create the misleading impression that Laos is attempting to propose an alternative mechanism to existing frameworks. This is not well-grounded for a small state.

 The author should recalibrate the analytical focus. Instead of portraying Laos as an actor that “creates” new frameworks, the paper should concentrate on how Laos interprets and implements the ASEAN Outlook on the Indo-Pacific (AOIP). The manuscript needs to clarify: How does Laos’s particular interpretation of AOIP generate challenges or opportunities in the implementation of this framework among other member states? Anchoring the analysis in official Lao diplomatic documents, rather than speculating about “new frameworks,” would enhance the credibility of the geopolitical analysis.

 General conclusion:

 The manuscript has shown improvement; however, issues related to data accuracy and the looseness of arguments regarding geopolitical roles remain significant obstacles. I recommend that the author carefully reassess all overly assertive claims and supplement them with concrete qualitative and/or quantitative evidence to substantiate the arguments presented above.

Is the topic of the opinion article discussed accurately in the context of the current literature?

Yes

Are arguments sufficiently supported by evidence from the published literature?

Partly

Are all factual statements correct and adequately supported by citations?

Partly

Are the conclusions drawn balanced and justified on the basis of the presented arguments?

Partly

Reviewer Expertise:

International Relations, Political Science, Historical Sciences

I confirm that I have read this submission and believe that I have an appropriate level of expertise to confirm that it is of an acceptable scientific standard, however I have significant reservations, as outlined above.

# Reviewer report: Reviewer response for version 2

**DOI:** 10.5256/f1000research.197347.r474704

**Published:** 2026-04-11

**Authors:** Tridib Chakraborti, Dr. Dona Ganguly

**Affiliations:** 1 Adamas University, Kolkata, West Bengal, India; 2 The Bhawanipur Education Society College, University of Calcutta, Kolkata, West Bengal, India

The article addresses important question of the role of Laos in ASEAN as well as its neutrality posture of a small state in context of China's ambitious strides in the region.

**Limitations and Areas of Improvement:**

1. Concept of neutrality should be analysed in context of hedging, band wagoning and balance of power.
2. The article seems to be self-contradictory in nature. On one hand, it claims that public opinion in Laos is showing increasing negative attitude towards China, on the other hand it also claims that Laos is an authoritarian state with limited scope for public opinion. The article has failed to address this ambiguity.
3. Emphasis should be made on the importance of Indo-Pacific in context of the foreign policy of Laos.
4. More empirical evidence is required to substantiate the content of the article.

5. The article seems to be more descriptive rather than analytical and citations are overweighing the actual arguments.

 6.  A more comprehensive theoretical analysis will be essential to enrich the article.

 7. Lack of primary sources and very few Laos Government’s official records make the article occasionally lacking in clarity.

Is the topic of the opinion article discussed accurately in the context of the current literature?

Yes

Are arguments sufficiently supported by evidence from the published literature?

Yes

Are all factual statements correct and adequately supported by citations?

Yes

Are the conclusions drawn balanced and justified on the basis of the presented arguments?

Partly

Reviewer Expertise:

NA

We confirm that we have read this submission and believe that we have an appropriate level of expertise to confirm that it is of an acceptable scientific standard, however we have significant reservations, as outlined above.

# Response

**Published:** 2026-04-13

**Authors:** bama andika putra

**Affiliations:** University of Bristol School of Sociology Politics and International Studies, Bristol, England, UK

Comment 1: Concept of neutrality should be analysed in context of hedging, band wagoning and balance of power.

 I refrain from taking a structural definition on neutrality for this article, considering that in the terminologies that Reviewer 4 mentions, there is an expected slight leaning towards one actor or both great powers. If I was to define it in the context of hedging, for example, the expectation would be that Laos is aiming to facilitate the interests of competing great powers (US and China) throughout its ASEAN chairmanship (which it did not). Rather, it solely took the route of defending ASEAN’s existing mechanisms. By neutrality, in this revised version, I explain that it entails Laos not taking sides in any great power conflict. The revised paragraph (page 2, paragraph 1): “Laos has held the Association of Southeast Asian Nations (ASEAN) chairmanship three times since joining the organization in 1997. ASEAN is a unique regional organization, prioritizing the importance of what scholars and observers name as the ‘ASEAN Way,’ with its diplomatic conduct geared to the non-coercive resolution of conflicts, consensus-based decision-making, and non-interference in the domestic politics of its member states ( Narine, 1997; Caballero-Anthony, 2005; Beeson, 2009; Tekunan, 2015; Darwis, Putra, and Cangara, 2020). In Laos’ chairmanships in 2016 and 2024, Laos displayed its neutrality (not taking the side of any great power), aiming to take the middle pathway in tensions that have divided the ASEAN members, such as the Indo-Pacific great power rivalry ( Saha, 2018; Tertia and Perwita, 2018; Medcalf, 2019; Mubah, 2019; Wheeler, 2020), and the South China Sea dispute ( Fravel, 2011; Thayer, 2011; Blazevic, 2012; Yahuda, 2013; Putra, 2020, 2022, 2023b; Nguyễn Anh, 2023). As a small, one-party, landlocked Southeast Asian state, how can we make sense of Laos’ neutrality?”

 Comment 2: The article seems to be self-contradictory in nature. On one hand, it claims that public opinion in Laos is showing increasing negative attitude towards China, on the other hand it also claims that Laos is an authoritarian state with limited scope for public opinion. The article has failed to address this ambiguity.

 The article acknowledges that Laos is technically a non-democratic nation, but also highlights the presence of negative sentiments from the Lao people. This study aims to provide an initial argumentation that neoclassical realism’s variables can help understand the empirical puzzle associated to why neutrality is displayed in Laos’ ASEAN chairmansip. As one of the intervening variables in the theory looks at the society and leader relations, it is inevitable to discuss the potential of the society perception variable in future studies assessing a neoclassical realism interpretation to the Laos foreign policy. Nevertheless, I acknowledge the reviewer 2’s point, and slightly changed the last paragraph of section 2: “The case of Laos is particularly interesting for this assessment, as its governance is confined to the authority of the LPRP, the dominant and only political party in Laos. As one of the few states that openly endorse communism and adopt authoritarian rule, it could, with ease, represent China’s national interests in ASEAN to secure more funding for its ambitious development plans ( Stuart-Fox, 1998; Cuyvers, 2019; Atkinson, 2021). Nevertheless, its consideration of potential consequences underscores the relevance of intervening variables that fuel Laos’ foreign policies, demonstrating that even non-democratic nations show a certain level of care for how society perceives critical issues affecting their daily lives.”

 Comment 3: Emphasis should be made on the importance of Indo-Pacific in context of the foreign policy of Laos.

 I agree that further discussions are needed on this. Nevertheless, I emphasize that the concern for Laos vis-à-vis the Indo-Pacific primarily relates to the undermining of ASEAN’s role in the region and the need to maintain ASEAN’s norms amid tensions between the Indo-Pacific great powers. The revised paragraph in page 4, paragraph 1: “In 2016, Laos remained neutral amid tensions between the Philippines and China over the language used in the 2016 Joint Statement of ASEAN Foreign Ministers and China. The Philippines, being a victim of China’s assertive claiming within its Exclusive Economic Zone, demanded that the chair include legal phrases in the joint statement; meanwhile, China preferred the adoption of vague language ( Odgaard, 2003; Sayalath and Creak, 2017; Storey, 2018; Hu, 2021; Kittikhoun, 2022; Sayalath, 2024). Laos chose the middle path and decided to make both parties equally unhappy by refraining from favoring either interest ( Lin, 2023b). As one of the joint statement’s operative clauses mentioned: “The parties reaffirm their respect for and commitment to the freedom of navigation in and overflight above the South China Sea as provided for by the universally recognized principles of international law” ( ASEAN, 2016, p. 2). This shows that Laos, vis-à-vis the Indo-Pacific, is mainly concerned with ASEAN's role and with maintaining its norms amid tensions involving Indo-Pacific powers.”

 Other comments: More empirical evidence is required to substantiate the content of the article; The article seems to be more descriptive rather than analytical and citations are overweighing the actual arguments; A more comprehensive theoretical analysis will be essential to enrich the article; Lack of primary sources and very few Laos Government’s official records make the article occasionally lacking in clarity.

 I would help if Reviewer 4 could clarify which parts of the article require more empirical evidence to substantiate the content. The discussions in section 3 follow neoclassical realism’s structure, which examines systemic and domestic variables. Implemented in the context of Laos’ foreign policy, this has been substantiated by the ASEAN Summit chair’s statements, several empirical cases related to the region’s dynamics and domestic perceptions towards China, and an assessment of Laos’ unique system. Meanwhile, data on primary sources considered for the study mainly comes from ASEAN statements (44th and 45th ASEAN Summits), which should reflect the decisiveness of Laos in maintaining neutral stances, compared to Cambodia’s 2012 ASEAN chairmanship. 

# Reviewer report: Reviewer response for version 2

**DOI:** 10.5256/f1000research.197347.r470021

**Published:** 2026-04-08

**Authors:** Dwi Ardhanariswari Sundrijo

**Affiliations:** 1 University of Indonesia, Depok City, Indonesia

The title and abstract suggest the article looks at how Laos behaved as ASEAN chair in its two most recent terms, especially given its close strategic and economic ties with China. The author describes Laos' approach as one of 'neutrality' and calls this an 'empirical anomaly', using Neoclassical Realism to explain it.

 Having read the manuscript, I see three main points for improvement. First, the idea of 'neutrality' needs more development. In general terms, neutrality means not taking sides in a conflict. Given the article's focus, we would expect to see how Laos manages competing pressures from China and ASEAN, especially as it relies on China economically while leading the region. However, apart from brief mentions of issues like the South China Sea, the article does not clearly set out what ASEAN's interests are, and how they might differ from China's. Without showing this tension, the claim that Laos is being 'neutral' feels unsupported.

 Second, calling this behaviour an 'empirical anomaly' - mainly by comparing it to Cambodia's chairmanship - seems too simple. It risks underestimating how much Laos values ASEAN and its role as chair. If the author wants to keep this point, they need to explain more clearly, with theoretical backing, why neutrality should not normally be expected in this situation, so that choosing it really does count as an anomaly.

 Third, using Neoclassical Realism does not mean ASEAN has to be left out of the analysis. States do not only balance or bandwagon; they also hedge, and regional bodies like ASEAN can be part of that strategy. As chair, a smaller state like Laos might prefer a light-touch approach to regionalism, but it can still use ASEAN's processes and norms to strengthen its position. This goes beyond the 'ASEAN Way' (non-interference, consensus) already mentioned, to include ideas like ASEAN Centrality and peaceful dispute settlement when dealing with partners outside the region.

 In short, if the ASEAN chairmanship is the main context for assessing Laos' foreign policy, the article needs to say more about what ASEAN itself wants and why it matters. This would help ground the 'neutrality' argument, make the 'anomaly' claim more convincing, and make the overall story easier to follow.

Is the topic of the opinion article discussed accurately in the context of the current literature?

Yes

Are arguments sufficiently supported by evidence from the published literature?

Yes

Are all factual statements correct and adequately supported by citations?

Partly

Are the conclusions drawn balanced and justified on the basis of the presented arguments?

Partly

Reviewer Expertise:

ASEAN Regionalism, Transnationalism, Postcolonialism, Norms Localization

I confirm that I have read this submission and believe that I have an appropriate level of expertise to confirm that it is of an acceptable scientific standard, however I have significant reservations, as outlined above.

# Response

**Published:** 2026-04-13

**Authors:** bama andika putra

**Affiliations:** University of Bristol School of Sociology Politics and International Studies, Bristol, England, UK

Comment 1: First, the idea of 'neutrality' needs more development. In general terms, neutrality means not taking sides in a conflict. Given the article's focus, we would expect to see how Laos manages competing pressures from China and ASEAN, especially as it relies on China economically while leading the region. However, apart from brief mentions of issues like the South China Sea, the article does not clearly set out what ASEAN's interests are, and how they might differ from China's. Without showing this tension, the claim that Laos is being 'neutral' feels unsupported.

 Response 1: I acknowledge that neutrality in the context of Laos’ ASEAN policy needs clarification. I have done this in the revision article, elaborating that neutrality in this context is by not siding to any great power. The revised paragraph (page 2, paragraph 1): “Laos has held the Association of Southeast Asian Nations (ASEAN) chairmanship three times since joining the organization in 1997. ASEAN is a unique regional organization, prioritizing the importance of what scholars and observers name as the ‘ASEAN Way,’ with its diplomatic conduct geared to the non-coercive resolution of conflicts, consensus-based decision-making, and non-interference of the domestic politics of its member states ( Narine, 1997; Caballero-Anthony, 2005; Beeson, 2009; Tekunan, 2015; Darwis, Putra, and Cangara, 2020). In Laos’ chairmanships in 2016 and 2024, Laos displayed its neutrality (not taking the side of any great power), aiming to take the middle pathway in tensions that have divided the ASEAN members, such as the Indo-Pacific great power rivalry ( Saha, 2018; Tertia and Perwita, 2018; Medcalf, 2019; Mubah, 2019; Wheeler, 2020), and the South China Sea dispute ( Fravel, 2011; Thayer, 2011; Blazevic, 2012; Yahuda, 2013; Putra, 2020, 2022, 2023b; Nguyễn Anh, 2023). As a small, one-party, landlocked Southeast Asian state, how can we make sense of Laos’ neutrality?”

 Response 2: Most of the explanations elaborating ASEAN’s interests can be seen in the first paragraph of section 3. The revised version emphasizes the words of interest, looking at how ASEAN aims to establish amity amid differences of opinions and interests among the nations at conflict in the South China Sea (and in general). The revised paragraph: “Laos’ ASEAN chairmanship in 2016 and 2024 faced several challenges rooted in China’s involvement in the region. By 2016, China’s maritime diplomatic strategies had evolved to a level that was perceived as assertive by claimant states to the South China Sea, the Philippines, Vietnam, Brunei Darussalam, and Malaysia ( Reuters, 2016; Yu, 2016; Basawantara, 2020; Chubb, 2022; Putra and Cangara, 2022; Putra, 2023a). The deepened great power politics in the Indo-Pacific also brought into question the possible fading role of ASEAN in the Indo-Pacific region as great powers started to establish their groupings to define, in their terms, the geopolitical significance of the region ( Chacko and Willis, 2018; Scott, 2019; Singh and Tsjeng, 2020; Tan, 2020; Hall, Lee-Brown and Strating, 2024). Such conditions undermine ASEAN’s interests, which aim to establish cooperative relations among actors in the region, so that disputes do not escalate in the future. Fortunately, there is a discourse showing that recently introduced initiatives such as the ASEAN Outlook on the Indo-Pacific (AOIP) have served as ASEAN s proactive geopolitical tool to shape Southeast Asia’s responses towards uncertainties deriving from great power tensions in alignment to the ASEAN Way ( Acharya, 2019; Anwar, 2020; Singh and Tsjeng, 2020; Putra, Cangara and Darwis, 2024). The AOIP, therefore, has served as a shield for ASEAN, protecting the regional organization from the intensification of the US-China rivalry through measures consistent with ASEAN’s principles.”

 Comment 2: Second, calling this behaviour an 'empirical anomaly' - mainly by comparing it to Cambodia's chairmanship - seems too simple. It risks underestimating how much Laos values ASEAN and its role as chair. If the author wants to keep this point, they need to explain more clearly, with theoretical backing, why neutrality should not normally be expected in this situation, so that choosing it really does count as an anomaly.

 : Neutrality here is understood as an empirical anomaly based on the comparison to Cambodia’s ASEAN chairmanship in 2012. The revised paragraph on page 2, paragraph 2, explains this, and uses Laos’ close alignment with China in recent years as a supportive argument to explain the empirical anomaly. The revisions: “Regarding China’s Belt and Road Initiative (BRI), the expectation is that Laos would align with China within ASEAN. Within the context of ASEAN chairmanship, Cambodia’s close economic ties with China led its 2012 chairmanship to lean toward China's interests in the South China Sea (Minh Vu, 2019; Dunst, 2021; Pich, 2021). Furthermore, studies have already concluded that Laos is a ‘vassal’ or ‘satellite’ nation to China ( Hunt, 2016; Kuik, 2021; Macan-Markar, 2022; Lin, 2023a; Mahtani and Huiying, 2024). China’s total investment in Laos from 2005 to 2024 amounts to USD 16.5 billion ( AEI, 2024). Several large-scale infrastructure development projects that Chinese investments have helped build since the launch of the BRI in 2013 include the Vientiane-Boten railway, special economic zones, and hydropower dams ( Kuik, 2021; LMOFA, 2021; Seneviratne, 2024). Consequently, scholars have argued for a strong connection between China’s BRI and the survival of the Lao People’s Revolutionary Party (LPRP) regime ( Lampton, Ho and Kuik, 2020; Kuik, 2021; Kuik and Rosli, 2023). Therefore, why doesn’t Laos display a similar gesture?”

 Comment 3: Third, using Neoclassical Realism does not mean ASEAN has to be left out of the analysis. States do not only balance or bandwagon; they also hedge, and regional bodies like ASEAN can be part of that strategy. As chair, a smaller state like Laos might prefer a light-touch approach to regionalism, but it can still use ASEAN's processes and norms to strengthen its position. This goes beyond the 'ASEAN Way' (non-interference, consensus) already mentioned, to include ideas like ASEAN Centrality and peaceful dispute settlement when dealing with partners outside the region.

 The revised article focuses the discourse on the fact that Laos is looking to place greater emphasis on existing mechanisms, as also suggested by Reviewer 3, but not aiming to shape the regional order through new mechanisms. Therefore, their actions are far from passive, as they embrace ASEAN’s interests. Several of the revisions are shown in section 3. The emphasis also aligns with your suggestions in highlighting ASEAN’s interests. 

 Paragraph 1: “Laos’ ASEAN chairmanship in 2016 and 2024 faced several challenges rooted in China’s involvement in the region. By 2016, China’s maritime diplomatic strategies had evolved to a level that was perceived as assertive by claimant states to the South China Sea, the Philippines, Vietnam, Brunei Darussalam, and Malaysia ( Reuters, 2016; Yu, 2016; Basawantara, 2020; Chubb, 2022; Putra and Cangara, 2022; Putra, 2023a). The deepened great power politics in the Indo-Pacific also brought into question the possible fading role of ASEAN in the Indo-Pacific region as great powers started to establish their groupings to define, in their terms, the geopolitical significance of the region ( Chacko and Willis, 2018; Scott, 2019; Singh and Tsjeng, 2020; Tan, 2020; Hall, Lee-Brown and Strating, 2024). Such conditions undermine ASEAN’s interests, which aim to establish cooperative relations among actors in the region, so that disputes do not escalate in the future. Fortunately, there is a discourse showing that recently introduced initiatives such as the ASEAN Outlook on the Indo-Pacific (AOIP) have served as ASEAN s proactive geopolitical tool to shape Southeast Asia’s responses towards uncertainties deriving from great power tensions in alignment to the ASEAN Way ( Acharya, 2019; Anwar, 2020; Singh and Tsjeng, 2020; Putra, Cangara and Darwis, 2024). The AOIP, therefore, has served as a shield for ASEAN, protecting the regional organization from the intensification of the US-China rivalry through measures consistent with ASEAN’s principles.”

 Paragraph 3: “Meanwhile, Laos’ 2024 chairmanship emphasized ASEAN centrality amid tensions in the Indo-Pacific. As stated in the 44 th and 45 th ASEAN Summit Chairman’s Statement, Laos reiterated the relevance of the AOIP for the region by reaffirming “ASEAN’s commitment to promote an enabling environment for peace, stability, and prosperity in the region by leading the evolving regional architecture including through ASEAN-led mechanisms and managing the impact of geopolitical and geostrategic shifts …” ( ASEAN, 2024, p. 10). With regards to the South China Sea dispute, Laos again refrained from siding with China by emphasizing the importance of an ASEAN-centered solution and parties to avoid confrontational actions: “We emphasized the importance of self-restraint in the conduct of all activities by claimants and all other states […] and thus welcomed practical measures that could reduce tensions and the risks of accidents, misunderstandings, and miscalculations” ( ASEAN, 2024, p. 41). Ultimately, Laos is showing that it places great trust in established frameworks rather than aiming to shape the regional order through new mechanisms.”

# Reviewer report: Reviewer response for version 2

**DOI:** 10.5256/f1000research.197347.r467548

**Published:** 2026-03-31

**Authors:** Mansur Juned

**Affiliations:** 1 Universitas Pembangunan Nasional Veteran Jakarta, Jakarta, Special Capital Region of Jakarta, Indonesia

I appreciate the author(s) taking the time to revise the manuscript based on my initial feedback. The revisions have certainly improved the paper. However, there are a few areas that require further refinement to ensure academic precision, particularly concerning my previous comments #2 and #5.

 Please address the following points:

 1. Refinement of Comment #2 (Laos’s Debt-to-GDP Ratio)

 While I note your updates regarding Laos's macroeconomic situation, the definitive claim that Laos has an "over 100% debt-to-GDP ratio" in 2024 still needs nuance. Because diverse macroeconomic trackers report slightly different values, stating definitively that it is "over 100%" can read as an overstatement.

 To strengthen your actual argument, I suggest revising this to acknowledge the statistical variance. You should emphasize that, regardless of the exact decimal, most authoritative sources agree the ratio is precariously high—around 100% of its GDP for 2024. Framing it this way avoids factual overstatement while reinforcing your core claim: that Laos's debt remains at a dangerous level, even when accounting for its recent declining rate. Additionally, please ensure this claim is backed by primary institutional citations, such as the IMF, World Bank, AMRO, Fitch, or Statista.

 2. Clarification of Comment #5 (Actively Shaping Regional Order & the AOIP)

 The current phrasing regarding how Laos is "actively shaping the regional order" risks misinterpretation. To clarify my previous comment, the argument should not imply that Laos is attempting to formulate another or alternative ASEAN Outlook on the Indo-Pacific (AOIP).

 Instead, please revise the text to focus on Laos's interpretation of the existing AOIP. The manuscript should articulate how Laos’s specific understanding of the AOIP creates new considerations for how the framework is actually implemented by other ASEAN Member States (AMS). Grounding Laos's diplomatic agency in its interpretation of established frameworks, rather than the creation of new ones, will make your geopolitical analysis much stronger and more accurate.

Is the topic of the opinion article discussed accurately in the context of the current literature?

Yes

Are arguments sufficiently supported by evidence from the published literature?

Partly

Are all factual statements correct and adequately supported by citations?

Partly

Are the conclusions drawn balanced and justified on the basis of the presented arguments?

Yes

Reviewer Expertise:

Area of the research: Security Studies, Cybersecurity, Geopolitic in Indo-Pacific

I confirm that I have read this submission and believe that I have an appropriate level of expertise to confirm that it is of an acceptable scientific standard, however I have significant reservations, as outlined above.

# Response

**Published:** 2026-04-13

**Authors:** bama andika putra

**Affiliations:** University of Bristol School of Sociology Politics and International Studies, Bristol, England, UK

Comment 1: Refinement of Comment #2 (Laos’s Debt-to-GDP Ratio)

 : Thank you for the recommendations. This point is revised and follows the wording suggested by Reviewer 2. The revisions (page 4, paragraph 4): “Structural modifiers are another systemic-stimuli variable relevant to Laos’ actions during its ASEAN chairmanships. Laos is a landlocked country, which ultimately means that its options for development are severely limited. Laos perceives it as pivotal to diversify its economic relations with Southeast Asian states by demonstrating good faith in ASEAN, thereby accelerating Laos’ integration into Southeast Asian markets. In its current form, Laos is categorized as part of the CMLV (Cambodia, Myanmar, Laos, Vietnam) group, which comprises the least-developed economies in Southeast Asia ( Chirathivat, 2002; Narjoko and Amri, 2007; Cuyvers, 2019). A display of commitment to the ASEAN Way allows Laos to explore alternative avenues of cooperation. The economic ties between Laos and China have negatively affected Laos’ foreign debt. Regardless of the exact decimal, most authoritarive sources agree the ratio is precariously high-around 100% of its GDP for 2024 ( Macan-Markar, 2022; Tiwari, 2024; Walker, 2024). Although the exact figures have been debated, there is still widespread recognition that Laos’ debts are concerning.”

 2. Clarification of Comment #5 (Actively Shaping Regional Order & the AOIP)

 : I have revised this point by emphasizing how Laos is supporting the implementation of established frameworks, rather than acting to shape regional order. As suggested, I also emphasize that Laos’ chairmanship is aiming to emphasize the need of adopting  a regional order based on what exists (AOIP). The revised paragraph (page 4, paragraph 2): “Meanwhile, Laos’ 2024 chairmanship emphasized ASEAN centrality amid tensions in the Indo-Pacific. As stated in the 44 th and 45 th ASEAN Summit Chairman’s Statement, Laos reiterated the relevance of the AOIP for the region by reaffirming “ASEAN’s commitment to promote an enabling environment for peace, stability, and prosperity in the region by leading the evolving regional architecture including through ASEAN-led mechanisms and managing the impact of geopolitical and geostrategic shifts …” ( ASEAN, 2024, p. 10). With regards to the South China Sea dispute, Laos again refrained from siding with China by emphasizing the importance of an ASEAN-centered solution and parties to avoid confrontational actions: “We emphasized the importance of self-restraint in the conduct of all activities by claimants and all other states […] and thus welcomed practical measures that could reduce tensions and the risks of accidents, misunderstandings, and miscalculations” ( ASEAN, 2024, p. 41). Ultimately, Laos is showing that it places great trust in established frameworks rather than aiming to shape the regional order through new mechanisms.”

# Reviewer report: Reviewer response for version 1

**DOI:** 10.5256/f1000research.194960.r456260

**Published:** 2026-02-23

**Authors:** Mansur Juned

**Affiliations:** 1 Universitas Pembangunan Nasional Veteran Jakarta, Jakarta, Special Capital Region of Jakarta, Indonesia

The author correctly identifies the current academic tension between those viewing Laos as a "vassal state" and those seeing a "balancing strategy". However, the literature review could be improved by more explicitly linking the ASEAN Way to the ASEAN Outlook on the Indo-Pacific (AOIP) as a proactive geopolitical tool rather than just a set of procedural norms

 Are all factual statements correct and adequately supported by citations?

 Assessment: Partially Yes.

 Detail: Key factual claims, such as the total volume of Chinese investment in Laos (USD 16.5 billion) and the country's debt-to-GDP ratio (over 100%), are well-supported by credible citations from the American Enterprise Institute and various regional reports but would differs significantly with the report from IMF, Fitch, AMRO, and Statista which put the debt to GDP ratio below 100%. Their is need to put the contrasting result on the manuscript while kept the main argument.

 Are arguments sufficiently supported by evidence from the published literature

 Assessment: Partially.

 Detail: The use of the neoclassical realism framework is well-grounded in foundational texts by Ripsman, Taliaferro, and Lobell. However, the argument that "state-society relations" (public sentiment) significantly drive foreign policy in a one-party autocratic state like the LPRP requires more robust evidence of the specific mechanism through which this sentiment influences the elite decision-makers.

 Additional Recommendation:

 1. Synthesize the "AOIP as a Shield": The author should more clearly argue that the AOIP is the geopolitical manifestation of the ASEAN Way. It is the tool Laos uses to translate "neutrality" into a functional regional architecture that resists being pulled into either the US or Chinese orbit.

 2. Put the highlight of how the intensification of the US-China rivalry is what forced the evolution of the AOIP to protect the "ASEAN Way".

 3 consider the idea that by using the AOIP, Laos isn't just "staying out of it," but is actively shaping a regional order where ASEAN remains the central manager of great power competition.

Is the topic of the opinion article discussed accurately in the context of the current literature?

Yes

Are arguments sufficiently supported by evidence from the published literature?

Partly

Are all factual statements correct and adequately supported by citations?

Partly

Are the conclusions drawn balanced and justified on the basis of the presented arguments?

Yes

Reviewer Expertise:

Area of the research: Security Studies, Cybersecurity, Geopolitic in Indo-Pacific

I confirm that I have read this submission and believe that I have an appropriate level of expertise to confirm that it is of an acceptable scientific standard, however I have significant reservations, as outlined above.

# Response

**Published:** 2026-03-03

**Authors:** bama andika putra

**Affiliations:** University of Bristol School of Sociology Politics and International Studies, Bristol, England, UK

COMMENT 1

 The author correctly identifies the current academic tension between those viewing Laos as a "vassal state" and those who see a "balancing strategy". However, the literature review could be improved by more explicitly linking the ASEAN Way to the ASEAN Outlook on the Indo-Pacific (AOIP) as a proactive geopolitical tool rather than just a set of procedural norms

 Response

 : I agree that the AOIP should be recognized as a proactive geopolitical tool introduced by ASEAN, which are consistent to the ASEAN Way. The literature review of this comment article focuses on the subject of Laos, and how it has navigated around the great power rivalries occurring within the region. Therefore, rather than place an explanation on the AOIP and how scholars have interpreted this as related to the ASEAN Way, this discourse is introduced after the literature review (page 3, first paragraph of section 3):

 “Laos’ ASEAN chairmanship in 2016 and 2024 faced several challenges rooted in China’s involvement in the region. By 2016, China’s maritime diplomatic strategies had evolved to a level that was perceived as assertive by claimant states to the South China Sea, the Philippines, Vietnam, Brunei Darussalam, and Malaysia ( Reuters, 2016; Yu, 2016; Basawantara, 2020; Chubb, 2022; Putra and Cangara, 2022; Putra, 2023a). The deepened great power politics in the Indo-Pacific also brought into question the possible fading role of ASEAN in the Indo-Pacific region as great powers started to establish their groupings to define, in their terms, the geopolitical significance of the region ( Chacko and Willis, 2018; Scott, 2019; Singh and Tsjeng, 2020; Tan, 2020; Hall, Lee-Brown and Strating, 2024). However, as noted in several other past studies, there is a discourse showing that recently introduced initiatives such as the ASEAN Outlook on the Indo-Pacific (AOIP) have served as ASEAN’s proactive geopolitical tool to shape Southeast Asia’s responses towards uncertainties deriving from great power tensions in alignment to the ASEAN Way (Acharya, 2019; Anwar, 2020; Singh and Tsjeng, 2020; Putra, Cangara and Darwis, 2024).f”

 COMMENT 2

 Are all factual statements correct and adequately supported by citations?

 Assessment: Partially Yes.

 Detail: Key factual claims, such as the total volume of Chinese investment in Laos (USD 16.5 billion) and the country's debt-to-GDP ratio (over 100%), are well-supported by credible citations from the American Enterprise Institute and various regional reports but would differs significantly with the report from IMF, Fitch, AMRO, and Statista which put the debt to GDP ratio below 100%. Their is need to put the contrasting result on the manuscript while kept the main argument.

 Response: On the point of Laos’ debt-to-GDP ratio, I am having contrasting results to those conveyed by the reviewer. Looking at AMRO and Statista, for example, I see that the figures are consistent with what I claim in the article (above 100%). Therefore, rather than introducing new figures into the argument, I chose to add a sentence at the end of page 4’s second last paragraph, which states that the exact figures are still debated. The revised paragraph:

 “Structural modifiers are another systemic-stimuli variable relevant to Laos’ actions during its ASEAN chairmanships. Laos is a landlocked country, which ultimately means that its options for development are severely limited. Laos perceives it as pivotal to diversify its economic relations with Southeast Asian states by demonstrating good faith in ASEAN, as this helps accelerate Laos’ integration into Southeast Asian markets. In its current form, Laos is categorized as part of the CMLV (Cambodia, Myanmar, Laos, Vietnam) group, which comprises the least-developed economies in Southeast Asia (Chirathivat, 2002; Narjoko and Amri, 2007; Cuyvers, 2019). A display of commitment to the ASEAN Way allows Laos to explore alternative avenues of cooperation. The economic ties between Laos and China have negatively affected Laos’ foreign debt, which is currently higher than 100% of its GDP (Macan-Markar, 2022; Tiwari, 2024; Walker, 2024). Although the exact figures have been debated, there is still a strong acknowledgement that Laos’ debts is concerning.”

 COMMENT 3

 Are arguments sufficiently supported by evidence from the published literature

 Assessment: Partially.

 Detail: The use of the neoclassical realism framework is well-grounded in foundational texts by Ripsman, Taliaferro, and Lobell. However, the argument that "state-society relations" (public sentiment) significantly drive foreign policy in a one-party autocratic state like the LPRP requires more robust evidence of the specific mechanism through which this sentiment influences the elite decision-makers.

 Response: There are multiple angles to respond to this inquiry. At the first level, I make clear that the neoclassical realism framework places importance on autocratic leaders having strong control over their state. However, I go on to explain that, as in the case of Cambodia, even in undemocratic settings, there is an immense potential for the people’s voices to be accommodated as a means to preserve power and ensure regime survival. I therefore conclude that although the LPRP has the means to exert power over its people, it does so in a way that still tries to accommodate the people's concerns, in order to preserve the regime and its survival. These three paragraphs below explain my point (revised):

 Point 1 in paragraph 1, page 5: “Assessing the unit and sub-unit variables of neoclassical realism poses one main challenge. Unlike in democratic settings, there is virtually no room for other voices of opposition to effect change within Laos’ governance due to its autocratic rule. Therefore, the LPRP holds the ultimate rule in the perception, decision-making, and policy implementation of its foreign policies.”

 Point 2 in paragraph 2, page 5: “Nevertheless, there is a chance for the LPRP to accommodate the growing voices calling for a distance from China's economic dependence. For example, as a comparison, studies have shown that even autocratic states consider the voices of their opposition. As seen in Cambodia, Hun Sen’s nearly three-decade rule had to start accommodating the concerns of opposition movements as the popularity of Cambodia’s opposition political party rose (Blanchard, 2017; Morgenbesser, 2019; Thul, 2023). Bader found that autocratic nations must display strong economic performance to compensate for deficiencies in political participation (Bader, 2015). However, if a growing opposition movement or voices are observed, the risks could be detrimental to a regime's survival.”

 Point 3 in paragraph 3, page 5: “Therefore, in the case of the LPRP, the state-society relations sub-unit variable under neoclassical realism is influential in understanding Laos’ accommodation of the negative sentiment opinions within Laos. If the LPRP were to choose to side with China, as Cambodia did during its ASEAN chairmanship in 2012, it would risk the perception of growing dependence on China. The Lao people are increasingly cautious of China’s investments within the state, so this would not be a strategic policy for the LPRP. Practically all of China’s BRI projects in Laos have faced public backlash. With the Vientiane-Boten railway project, for example, citizens questioned whether Laos was truly becoming the ‘hub’ of mainland Southeast Asian trade due to insufficient commodities to export ( Freeman, 2019; Seneviratne, 2024) and the fact that mining concessions were used as collateral in case the railway did not generate enough return for China’s investments ( Pang, 2017; SIIS, 2017; Albert, 2019; Walker, 2024). By taking the side of the ASEAN Way, the LPRP benefits by distancing itself from the discourse of being overly attached to China, thereby satisfying the Lao people’s concerns about injustices associated with the BRI projects in Laos.” 

 COMMENT 4:

 Synthesize the "AOIP as a Shield": The author should more clearly argue that the AOIP is the geopolitical manifestation of the ASEAN Way. It is the tool Laos uses to translate "neutrality" into a functional regional architecture that resists being pulled into either the US or Chinese orbit. It highlights that the intensification of the US-China rivalry forced the evolution of the AOIP to protect the "ASEAN Way".

 Response:

 I agree that the AOIP’s significance can be elevated in the texts. The revisions can be seen in page 3, paragraph 1 of section 3: “Laos’ ASEAN chairmanship in 2016 and 2024 faced several challenges rooted in China’s involvement in the region. By 2016, China’s maritime diplomatic strategies had evolved to a level that was perceived as assertive by claimant states to the South China Sea, the Philippines, Vietnam, Brunei Darussalam, and Malaysia ( Reuters, 2016; Yu, 2016; Basawantara, 2020; Chubb, 2022; Putra and Cangara, 2022; Putra, 2023a). The deepened great power politics in the Indo-Pacific also brought into question the possible fading role of ASEAN in the Indo-Pacific region as great powers started to establish their groupings to define, in their terms, the geopolitical significance of the region ( Chacko and Willis, 2018; Scott, 2019; Singh and Tsjeng, 2020; Tan, 2020; Hall, Lee-Brown and Strating, 2024). However, as noted in several other past studies, there is a discourse showing that recently introduced initiatives such as the ASEAN Outlook on the Indo-Pacific (AOIP) have served as ASEAN’s proactive geopolitical tool to shape Southeast Asia’s responses towards uncertainties deriving from great power tensions in alignment to the ASEAN Way (Acharya, 2019; Anwar, 2020; Singh and Tsjeng, 2020; Putra, Cangara and Darwis, 2024). The AOIP, therefore, has served as a shield for ASEAN, protecting the regional organization from the intensification of the US-China rivalry through measures consistent with ASEAN’s principles.”

 COMMENT 5

 3 consider the idea that by using the AOIP, Laos isn't just "staying out of it," but is actively shaping a regional order where ASEAN remains the central manager of great power competition.

 Response: I refrained from using the words ‘actively shaping a regional order’ in the context of Laos’ chairmanship role in ASEAN. I argue that, regarding the AOIP, Indonesia displayed the most agency in establishing the document among ASEAN member states. Therefore, Laos’ chairmanship in 2024 was simply to continue asserting the importance of abiding by the AOIP, rather than create new initiatives to shape the regional response through initiating new methods. I would use these words, if, for example, Indonesia contributed directly to the formation of the AOIP or forums/partnerships that were born from the AOIP (for example, the ASEAN Indo-Pacific Forum).

# Reviewer report: Reviewer response for version 1

**DOI:** 10.5256/f1000research.194960.r456254

**Published:** 2026-02-20

**Authors:** Quoc Dung Mai

**Affiliations:** 1 Ho Chi Minh City University of Industry and Trade, Ho Chi Minh City, Ho Chi Minh City, Vietnam

1. General Assessment

 The article addresses a highly compelling research problem with significant practical value: decoding Laos' "neutral" policy during its ASEAN Chairmanship (2016, 2024) despite the country’s profound economic dependence on China. The author successfully employs the Neoclassical Realism framework to challenge the prevailing view that Laos is merely a "vassal state."

 2. Key Strengths

 Appropriate Theoretical Framework: The application of Neoclassical Realism is a highly pertinent choice. It allows for an analysis of how "domestic filters" (intervening variables)—such as leadership perceptions and state-society relations—mediate and calibrate systemic pressures from the international environment.

 Sharp Insight into Internal Politics: The paper correctly identifies that even within a single-party system like Laos, the LPRP must account for public dissatisfaction regarding public debt and Belt and Road Initiative (BRI) projects to maintain political legitimacy.

 Empirical Evidence: The author provides concrete examples of how Laos coordinated ASEAN joint statements to avoid overt side-taking, thereby preserving the "ASEAN Way" and diversifying its foreign relations.

 3. Limitations and Areas for Improvement

 Despite these contributions, several points require further clarification and strengthening:

 Primary Data Gap: While the theoretical framework emphasizes "decision-maker perceptions," the article relies heavily on third-party surveys (e.g., ISEAS) and media reports. The author should incorporate more primary sources or a deeper analysis of official statements from Lao leadership to demonstrate how this "filtering" mechanism operates in practice.

 Weight of Variables: The paper does not clearly decouple the influence of historical factors (the special relationship with Vietnam) from current economic pressures from China. Clarifying which variable carries more weight at specific junctures would make the argument more persuasive.

 Insufficient Comparative Analysis: The comparison with Cambodia’s 2012 chairmanship remains somewhat superficial. Laos is a landlocked nation, which creates structural constraints in security and economic logistics that are vastly different from Cambodia's. The author should delve deeper into these geopolitical nuances.

 Monolithic View of Leadership: The article treats the LPRP as a monolithic entity in decision-making. A comprehensive Neoclassical Realist analysis should consider whether there are diverging views or factions within the elite regarding dependency on China.

 4. Conclusion

 This is a high-quality study that offers a fresh perspective on the foreign policy of small states in the region. The paper would gain significantly more weight if the author strengthens the empirical data regarding internal decision-making processes and deepens the comparative geopolitical analysis.

Is the topic of the opinion article discussed accurately in the context of the current literature?

Yes

Are arguments sufficiently supported by evidence from the published literature?

Partly

Are all factual statements correct and adequately supported by citations?

Partly

Are the conclusions drawn balanced and justified on the basis of the presented arguments?

Partly

Reviewer Expertise:

International Relations, Political Science, Historical Sciences

I confirm that I have read this submission and believe that I have an appropriate level of expertise to confirm that it is of an acceptable scientific standard, however I have significant reservations, as outlined above.

# Response

**Published:** 2026-03-03

**Authors:** bama andika putra

**Affiliations:** University of Bristol School of Sociology Politics and International Studies, Bristol, England, UK

COMMENT 1

 Primary Data Gap: While the theoretical framework emphasizes "decision-maker perceptions," the article relies heavily on third-party surveys (e.g., ISEAS) and media reports. The author should incorporate more primary sources or a deeper analysis of official statements from Lao leadership to demonstrate how this "filtering" mechanism operates in practice.

 Response: 

 Several primary sources are utilized in this comment article. Most notably, as the intention is to display the neutrality of Laos during its ASEAN chairmanships, the referenced sources are those that have been expresse during ASEAN forums, which includes in the 2016 and 2024 chairmanships. Several changes can be seen in page 3 (last paragraph) and 4 (first paragraph):

 Quote 1: As one of the joint statement’s operative clauses mentioned: “The parties reaffirm their respect for and commitment to the freedom of navigation in and overflight above the South China Sea as provided for by the universally recognized principles of international law” ( ASEAN, 2016, p.2).

 Quote 2: As stated in the 44 th and 45 th ASEAN Summit Chairman’s Statement, Laos reiterated the relevance of the ASEAN Outlook on the Indo-Pacific (AOIP) for the region by reaffirming “ASEAN’s commitment to promote an enabling environment for peace, stability, and prosperity in the region by leading the evolving regional architecture including through ASEAN-led mechanisms and managing the impact of geopolitical and geostrategic shifts …” ( ASEAN, 2024, p.10).

 Quote 3: With regards to the South China Sea dispute, Laos again refrained from siding with China by emphasizing the importance of an ASEAN-centered solution and parties to avoid confrontational actions: “We emphasized the importance of self-restraint in the conduct of all activities by claimants and all other states […] and thus welcomed practical measures that could reduce tensions and the risks of accidents, misunderstandings, and miscalculations” ( ASEAN, 2024, p.41).

 COMMENT 2

 Weight of Variables: The paper does not clearly decouple the influence of historical factors (the special relationship with Vietnam) from current economic pressures from China. Clarifying which variable carries more weight at specific junctures would make the argument more persuasive.

 Response: In page 4, paragraph 2, I have clarified what I mean on the point of significant historical factors that shape the Laos-Vietnam bilateral relations. Nevertheless, I refrain from using the terms ‘weighing’ as the neoclassical realism framework does not delve deep into comparing the significance of alliance relations as a response to systemic stimuli. The revised paragraph within the text:

 “The international system does not generate a fixed signal. China’s rise in the Asia-Pacific exerts pressure on smaller states like Laos to align with it to secure economic benefits. However, this is not an automatic process. Given the strategic environment of Laos, there remains a special perception reserved for Thailand and Vietnam (Thayer, 1982; Giang and Phuong, 2024; Phoonphongphiphat, 2024) due to historical attachments, convergence of political views, and mutual economic benefits. With Vietnam, for example, the special relationship can be attributed to the shared communist ideology and close ties that were earlier established during the Cold War. Fully siding with China will expose Laos to self-isolation and an overly dependent foreign policy. For Laos, over-dependence on a great power is dangerous to the state's stability, as seen in Laos’ struggle during the Cold War after the weakening and eventual collapse of the Soviet Union (Meng, 1987; Evans, 1998; Rathie, 2017; Leon, 2024).”

 COMMENT 3

 Insufficient Comparative Analysis: The comparison with Cambodia’s 2012 chairmanship remains somewhat superficial. Laos is a landlocked nation, which creates structural constraints in security and economic logistics that are vastly different from Cambodia's. The author should delve deeper into these geopolitical nuances.

 Response: The introduction of Cambodia into the analysis has the function of showing readers how another new member of ASEAN has shown that it can deviate away from the ASEAN Way when there are economic pressures that the state faces. In the context of Cambodia, as explained in the texts, the primary concern lies on the issue that both Cambodia and Laos are facing significant economic hardships, and this is somewhat contributed by the increasing debt incurred from China. As pointed, therefore in the analysis, the expectation is that Laos would display similar foreign policies to Cambodia during its chairmanships, due to the similarities of challenges encountered. This point is emphasized continuously:

 Page 2 paragraph 2: “Regarding China’s Belt and Road Initiative (BRI), the expectation is that Laos would align with China within ASEAN. Studies have already concluded that Laos is a ‘vassal’ or ‘satellite’ nation to China ( Hunt, 2016; Kuik, 2021; Macan-Markar, 2022; Lin, 2023a; Mahtani and Huiying, 2024). China’s total investment in Laos from 2005 to 2024 amounts to USD 16.5 billion (AEI, 2024). Several large-scale infrastructure development projects that Chinese investments have helped build since the launch of the BRI in 2013 include the Vientiane-Boten railway, special economic zones, and hydropower dams (Kuik, 2021; LMOFA, 2021; Seneviratne, 2024). Consequently, scholars have argued for a strong connection between China’s BRI and the survival of the Lao People’s Revolutionary Party (LPRP) regime (Lampton, Ho and Kuik, 2020; Kuik, 2021; Kuik and Rosli, 2023). In contrast to Laos’ position, Cambodia’s ASEAN chairmanship in 2012 aligned with China’s national interests regarding ASEAN’s response to the South China Sea (Minh Vu, 2019; Dunst, 2021; Pich, 2021). So why doesn’t Laos display a similar gesture?”

 Page 2, paragraph 3: “Therefore, in the case of the LPRP, the state-society relations sub-unit variable under neoclassical realism is influential in understanding Laos’ accommodation of the negative sentiment opinions within Laos. If the LPRP were to choose to side with China, as Cambodia did during its ASEAN chairmanship in 2012, it would risk the perception of growing dependence on China”

 COMMENT 4

 Monolithic View of Leadership: The article treats the LPRP as a monolithic entity in decision-making. A comprehensive Neoclassical Realist analysis should consider whether there are diverging views or factions within the elite regarding dependency on China.

 Response: I agree that this is a potential argument/interpretation in regards to Laos’ foreign policy. However, I have pre-emptively responded to this, as I explain the patterns of foreign policies in states that have questioned democratic practices in place. Most notably this is responded to in paragraph 2, page 5:

 “Nevertheless, accommodating the growing voices calling for a distance from China's economic dependence is heard within the LPRP. As studies have shown, even autocratic states consider the voices of their opposition. As seen in Cambodia, Hun Sen’s nearly three-decade rule had to start accommodating the concerns of opposition movements as the popularity of Cambodia’s opposition political party rose (Blanchard, 2017; Morgenbesser, 2019; Thul, 2023). Bader found that autocratic nations must display strong economic performance to compensate for deficiencies in political participation (Bader, 2015). However, if a growing opposition movement or voices are observed, the risks could be detrimental to a regime's survival.”
